# Mechanical roles of apical constriction, cell elongation, and cell migration during neural tube formation in *Xenopus*

**DOI:** 10.1007/s10237-016-0794-1

**Published:** 2016-05-18

**Authors:** Yasuhiro Inoue, Makoto Suzuki, Tadashi Watanabe, Naoko Yasue, Itsuki Tateo, Taiji Adachi, Naoto Ueno

**Affiliations:** 1 Institute for Frontier Medical Sciences, Kyoto University, Kyoto, Japan; 2 National Institute for Basic Biology, Okazaki, Aichi Japan

**Keywords:** Neural tube closure, 3D vertex simulation, Apical constriction, Cell elongation, Cell migration

## Abstract

Neural tube closure is an important and necessary process during the development of the central nervous system. The formation of the neural tube structure from a flat sheet of neural epithelium requires several cell morphogenetic events and tissue dynamics to account for the mechanics of tissue deformation. Cell elongation changes cuboidal cells into columnar cells, and apical constriction then causes them to adopt apically narrow, wedge-like shapes. In addition, the neural plate in *Xenopus* is stratified, and the non-neural cells in the deep layer (deep cells) pull the overlying superficial cells, eventually bringing the two layers of cells to the midline. Thus, neural tube closure appears to be a complex event in which these three physical events are considered to play key mechanical roles. To test whether these three physical events are mechanically sufficient to drive neural tube formation, we employed a three-dimensional vertex model and used it to simulate the process of neural tube closure. The results suggest that apical constriction cued the bending of the neural plate by pursing the circumference of the apical surface of the neural cells. Neural cell elongation in concert with apical constriction further narrowed the apical surface of the cells and drove the rapid folding of the neural plate, but was insufficient for complete neural tube closure. Migration of the deep cells provided the additional tissue deformation necessary for closure. To validate the model, apical constriction and cell elongation were inhibited in *Xenopus laevis* embryos. The resulting cell and tissue shapes resembled the corresponding simulation results.

**Electronic supplementary material:**

The online version of this article (doi:10.1007/s10237-016-0794-1) contains supplementary material, which is available to authorized users.

## Introduction

The neural tube is the anlage of the central nervous system (CNS) that gives rise to the brain and spinal cord, which are essential organs in animals that govern the sensing of environmental cues and control motion and behavior. During the development of amphibians, amniotes, and mammals, the neural tube is stereotypically formed from a flat sheet of neural epithelium (the neural plate) (Schoenwolf and Smith 1990; Colas and Schoenwolf 2001; Suzuki et al. 2012). The morphogenetic event known as neural tube closure is achieved through a bending of the plate and rolling up of both lateral boundaries with the non-neural epithelium (neural folds), which are then fused at the midline. In contrast, teleost fish adopt a slightly different mechanism in which neural progenitor cells migrate toward the midline from both lateral sides, and a mass of these cells accumulating at the midline forms a rod-like structure (the neural keel) in which a central lumen is eventually formed (Lowery and Sive 2004; Clarke 2009). Such variations among mechanisms of neural tube formation in different animals highlight the diversification of strategies for organ development during animal evolution (Harrington et al. 2009).

Recent studies using the embryos of an amphibian, *Xenopus laevis*, in which neural tube formation can be observed directly under a light microscope, have accumulated a significant amount of knowledge of the molecular and cellular mechanisms controlling neural tube formation. The ectoderm in *X. laevis* is a bilayer, comprising of superficial and deep layers. The formation of the tube structure from a sheet requires three physical events that are involved in cell morphogenesis and tissue dynamics (Suzuki et al. 2012). The most extensively studied cellular morphogenetic event is apical constriction, in which superficial neural cells in the neural plate accumulate F-actin on their apical side to form a thick F-actin band. The cell surface is minimized by the constriction of the actomyosin-based F-actin ring, leading to a change from a columnar shape into an apically narrow wedge-like shape (Schroeder 1970; Haigo et al. 2003; Lee et al. 2007). Concomitantly, cell elongation, in which the cell length (height) increases in the apico-basal (AB) direction, occurs in cells undergoing apical constriction (Lee et al. 2007; Suzuki et al. 2010). These cell shape changes are thought to occur near the midline, thereby generating forces that bend the neural plate and bring the two lateral neural folds together for closure (Suzuki et al. 2012); however, little is known about how these shape changes contribute to complete tube closure. Recently, we showed that cell migration of the non-neural ectoderm in the deep layer, which itself does not give rise to the neural tube, also contributes to complete closure by pulling on the two layers of the non-neural cell sheet to bring them to the midline (Morita et al. 2012). These findings suggest that neural tube closure is a complex process in which three physical events, including apical constriction, cell elongation, and cell migration, play key mechanical roles.

In the present study, we investigated whether a combination of these three physical events is mechanically sufficient to induce neural tube formation in *X.laevis*. Computational simulations have successfully recapitulated cellular motions based on force balances among cells and have been used to test hypotheses suggested by biological experiments (Honda et al. 2008a, b; Eiraku et al. 2011; Okamoto et al. 2013; Hirashima 2014). Here, a three-dimensional (3D) vertex model (Honda et al. 2004; Okuda et al. 2013a, 2015) is employed to perform computer simulations of different combinations of physical events in both control and inhibition models in silico to investigate how the three physical events contribute mechanically to the process of neural tube closure. In addition, simulation results obtained using the 3D vertex model can be compared directly with the experimental results. We assessed the validity of the simulations in reproducing the role of each physical event by comparing the results obtained from in silico analyses with those from in vivo experiments on embryos after molecular inhibition of the physical events.

## Modeling dynamics of the neural plate

### 3D vertex model expressing multicellular dynamics

The 3D vertex model represents the shape of a cell as a polyhedron consisting of vertices and edges. The tissue is represented as an aggregate of multiple cells, where the vertices and edges of each cell are shared by the neighboring cells (polyhedra). These vertices and edges compose a network that represents the entire shape of the tissue. Cell rearrangement is expressed by reconnecting local network patterns based on the reversible network reconnection rule (Okuda et al. 2013a).

**Table 1:** Model parameters: values in the bracket ($\cdot$) for inhibition models

Symbol	Value	Descriptions
Physical parameters of the energy functions		
$k^\mathrm{v}$	80	Volume elasticity of Eq. (4)
$k^\mathrm{s}$	1.0	Area elasticity of Eq. (5)
$k^\mathrm{h}$	$4.0\times 10^{-2}$; (0.0)	Height elasticity of elongating cell of Eq. (6)
$k^\mathrm{ac}$	$4.0\times 10^{-1}$; (0.0)	Apical circumference elasticity of Eq. (7)
$f_\mathrm{cm}$	$4.0\times 10^{-4}$; (0.0)	Driving force of cell migration of Eq. (8)
$v^\mathrm{eq}$	1.0	Cell volume in stress-free state of Eq. (4), $s^\mathrm{{eq}}$, and $l^\mathrm{{eq}}$
$s^\mathrm{eq}$	$2v^\mathrm{eq}/h^\mathrm{eq}+2\sqrt{2\sqrt{3}v^\mathrm{eq}h^\mathrm{eq}}$	Cell surface area (hexagonal prism) in stress-free state of Eq. (5)
$h^\mathrm{eq}$	1.0, 3.0; (1.0, 1.5, 2.0, 2.5)	Original and elongated cell height in stress-free state of Eq. (6), $s^\mathrm{{eq}}$, and $l^\mathrm{{eq}}$
$l^\mathrm{eq}$	$2\gamma ^\mathrm{cs}\sqrt{\pi v^\mathrm{eq}/h^\mathrm{eq}}$	Apical circumference length in stress-free state of Eq. (7)
$\gamma ^\mathrm{cs}$	0.90	Constriction scaling factor in $l^\mathrm{{eq}}$
Numerical parameters for computational simulations		
$\eta$	1.0	Friction coefficient of vertex of Eq. (1)
$\Delta t$	$1.0\times 10^{-3}$	Time step size for numerical integration of Eq. (1)
$\Delta t_\mathrm{{r}}$	1.0	Time interval at which the network reconnection rule is attempted
$\Delta l_\mathrm{{th}}$	$1.0\times 10^{-2}$	Threshold edge length, below which a local network is reconnected

The equation of motion of vertex *i* of which position vector ${\mathbf {r}}_i$ at time *t* is described as:

1 $$\begin{aligned} \eta \left( \frac{\mathrm{d}{} \mathbf{r}_{i}}{\mathrm{d}t}-\mathbf{V}_{i}\right) = -\nabla _{i} U. \end{aligned}$$

Here, $\eta$ is a friction coefficient and ${\mathbf {V}}_i$ is the mean velocity vector around vertex *i*. To satisfy Galilean invariance in vertex motion, the friction force of vertex *i* is defined in a local velocity frame (Okuda et al. 2015). In the 3D vertex model, vertex *i* is directly connected to four vertices by edges. Indexing these directly connected vertices as *j*(*i*), the mean velocity vector can be defined in the simplest form as:

2 $$\begin{aligned} {\mathbf {V}}_i= & {} \frac{1}{5}\left( \frac{\mathrm{d}{\mathbf {r}}_{i}}{\mathrm{d}t}+\sum _{j(i)}\frac{\mathrm{d}{\mathbf {r}}_{j(i)}}{\mathrm{d}t}\right) \text{. } \end{aligned}$$

The right-hand side of Eq. (1) indicates a force exerted on vertex *i* derived from the total energy function *U*, a function of the vertex positions that represents the mechanical properties and morphogenetic events of the cells as:

3 $$\begin{aligned} U= & {} \sum _{j^c}^{\mathrm{cell}} u_{j^c}^\mathrm{v} + u_{j^c}^\mathrm{s} + u_{j^c}^\mathrm{h} + u_{j^c}^\mathrm{ac} + u_{j^c}^\mathrm{cm} \text{, } \end{aligned}$$

where $\sum _{j^c}^{\mathrm{cell}}$ indicates a summation over all of the cells. Because vertex *i* is shared by multiple neighboring cells, energy functions of an arbitrary cell including vertex *i* contribute to the force exerted on vertex *i*. The energy function *U* includes the cell volume elastic energy $u_{j^c}^\mathrm{v}$, cell surface elastic energy $u_{j^c}^\mathrm{s}$, cell height elastic energy $u_{j^c}^\mathrm{h}$, apical constriction energy $u_{j^c}^\mathrm{ac}$, and cell migration energy $u_{j^c}^\mathrm{cm}$. The mathematical expressions for these energy functions are defined as:

4 $$\begin{aligned} u_{j^c}^\mathrm{v}\left( v_{j^c}\right)= & {} \frac{1}{2}k^{\mathrm{v}}\left( \frac{v_{j^c}}{v_{j^c}^{\mathrm{eq}}}-1\right) ^2 \text{, }\end{aligned}$$

5 $$\begin{aligned} u_{j^c}^\mathrm{s}\left( s_{j^c}\right)= & {} \frac{1}{2}k^{\mathrm{s}}\left( \frac{s_{j^c}}{s_{j^c}^{\mathrm{eq}}}-1\right) ^2 \text{, } \end{aligned}$$

6 $$\begin{aligned} u_{j^c}^\mathrm{h}\left( h_{j^c}\right)= & {} \frac{1}{2}k^{\mathrm{h}}\left( \frac{h_{j^c}}{h_{j^c}^{\mathrm{eq}}}-1\right) ^2 \text{, } \end{aligned}$$

7 $$\begin{aligned} u_{j^c}^\mathrm{ac}\left( l_{j^c}\right)= & {} \frac{1}{2}k^{\mathrm{ac}}\left( \frac{l_{j^c}}{l_{j^c}^\mathrm{eq}}-1\right) ^2 \text{, } \end{aligned}$$

8 $$\begin{aligned} u_{j^c}^\mathrm{cm}\left( x_{j^c}\right)= & {} f_\mathrm{cm}x_{j^c} \text{. } \end{aligned}$$

These mathematical expressions, except for $u_{j^c}^\mathrm{cm}$, were derived from a 3D vertex model of epithelial cells (Okuda et al. 2013a, b). The $j^c$-th cell’s volume $v_{j^c}$, surface area $s_{j^c}$, height $h_{j^c}$, apical circumferential length $l_{j^c}$, and position from the midline along the medial-lateral (ML) axis $x_{j^c}$ are represented as variables. The superscript eq in several variables in Eqs. (4–7) indicates the value in the stress-free state. The constants $k^\mathrm{v}$, $k^\mathrm{s}$, $k^\mathrm{h}$ and $k^\mathrm{ac}$ are the volume elasticity, surface elasticity, height elasticity, and apical circumference ring elasticity of the cell, respectively. Equation (7) is included in Eq. (3) only when the circumference ring is extended, that is, when $l_{j^c} > l_{j^c}^\mathrm{eq}$, contributing to a shortening of the circumferential length. The constant $f_\mathrm{cm}$ is a driving force for non-neural cell migration.

Constructing an arbitrary energy function around the local minimum energy, the energy function should be expressed by the second-order term of geometrical parameters, such as strain. This is because the first-order term violates the local minimum assumption. Therefore, the mathematical form of energy functions, Eqs. (4)–(7), are the same as those previously reported (Okuda et al. 2013b), but we assigned different values of each model constant (Table 1) from the previous study. The effect of altering this model constant is reported in “Appendix.”

### Simulation models

#### Simulation region and combination of physical events

**Fig. 1:**
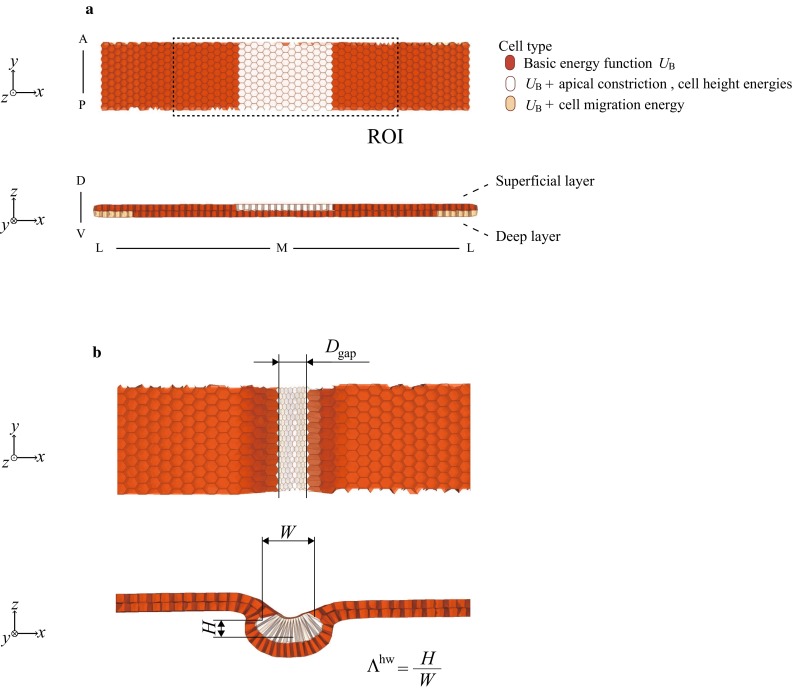
**a** Initial shape of the double-layered ectoderm for simulations. The neural and non-neural cells are hexagonally packed in superficial and deep layers, with the superficial neural cells displayed in *white*. The basic energy function $U_B$ includes the cell volume elastic energy $u_k^\mathrm{{v}}$ and cell surface elastic energy $u_k^\mathrm{{s}}$. The region of interest (ROI) is defined by the dashed rectangular region for visualization of the subsequent figures. *A* anterior, *P* posterior, *M* midline, *L* lateral side, *D* dorsal, *V* ventral. **b** Characteristic geometrical parameters on the tissue scale. The gap distance, $D_\mathrm{gap}$, is the mean end-to-end distance between the two centroids of the apical surfaces on the lateral boundary cells of the neural plate. The tissue height *H* and width *W* of the neural plate are defined by the differences between the largest and smallest *z*- and *x*-components of the position vector of the neural cell centroid. Then, the tissue height/width ratio $\Lambda _\mathrm{{hw}}$ is defined as $\Lambda _\mathrm{{hw}}=H/W$

To investigate the tissue deformations during neural tube closure using simulations, we focused on a rectangular strip of the ectoderm as the simulated region and not the full embryo (Fig. 1a), in which the Cartesian coordinate system (*x*, *y*, *z*) was defined along the ML, anterior–posterior (AP), and dorsal-ventral (DV) axes. Based on experimental observations of *X. laevis* embryos described below, approximately 15 neural cells reside along the ML axis in both the superficial and deep layers, composing a neural plate. These neural cells are bounded laterally by 22 non-neural cells on either side in each layer. Thus, the size of the strip in the ML direction is 59 cells in each layer. The size of the strip in the AP direction is 10 cells, with a periodic boundary condition.

**Table 2:** Simulation models showing activation (+) and inhibition (−) of each physical event

Model	Apical constriction	Cell elongation	Cell migration
I	+	−	−
II	+	+	−
III	+	+	+
IV	−	−	+
V	+	−	+

We used five simulation models described below, which were characterized by different combinations of physical events (Table 2), including apical constriction, cell elongation, and cell migration. The control model includes all three events, represented by model III. The remaining models are inhibition models constructed by changing the parameter values of the energy functions relative to those in the control model. All simulations start from an equilibrated configuration of vertices obtained by minimizing a basic energy function, which includes the cell volume elastic energy and cell surface elastic energy.

Neural and non-neural cells in the stress-free state are assumed to be regular hexagonal columnar shapes. Based on this assumption, the reference surface $s^\mathrm{{eq}}$ is expressed by the reference volume $v^\mathrm{{eq}}$ and height $h^\mathrm{{eq}}$ of the cell given in Table 1. Thus, there is a redundancy in the energy functions; however, the contribution of the cell surface elastic energy on the cell height is rather passive, while the cell height elastic energy is required to express an active cell elongation (Lee et al. 2007; Suzuki et al. 2010). Further discussion is given in “Appendix,” along with the surveying parameters.

The simulation time *t* displayed for the results is normalized by the duration of neural tube closure that occurs in the control model. In this study, we focused on neurulation from stages 13 to 19, in which the elapsed times from stage 13 to stages 15, 16, and 19 are estimated as 164 min, 210 min, and 360 min, respectively (Nieuwkoop and Faber 1967). Consequently, the simulation time points $t=$ 0.0, 0.46, 0.58, and 1.0 are introduced as a rough indication of stages 13, 15, 16, and 19, respectively.

#### Apical constriction

Apical constriction is a relatively common morphogenetic event that occurs in a variety of developmental processes in multicellular organisms (Sawyer et al. 2010). Actomyosin contractility causes a drastic shrinkage of the apical surface area of each cell and leads to a well-known change in cell morphology from a columnar to a wedge-like shape (Schroeder 1970; Haigo et al. 2003; Lee et al. 2007). Based on experimental observations (Suzuki et al. 2012), only neural cells in the superficial layer undergo apical constriction. The onset of apical constriction is likely an intermediate time point between stages 15 and 16 (Lee et al. 2007). Thus, in our simulations, apical constriction is defined by Eq. (7) and acts on all of the superficial neural cells starting at time $t=0.49$ (corresponding to an intermediate time point between stages 15 and 16).

#### Cell elongation

The neural plate thickens in the AB direction during neural tube formation (Schroeder 1970; Davidson and Keller 1999; Suzuki et al. 2012). In the present study, for simplicity, we took into account only cell elongation induced in the superficial layer. Because superficial neural cells undergo a drastic elongation that precedes apical constriction by one or two stages (Lee et al. 2007), a change in cell height was imposed on the superficial neural cells starting at the beginning of the simulation. On activation of cell elongation, cell height $h^{\mathrm{eq}}$ in the stress-free state tripled and the cell height elastic energy was imposed.

#### Cell migration

Although the non-neural epithelium does not contribute to the neural tube itself, it does play a role during neural tube closure, suggesting a non-tissue autonomous mechanism (Morita et al. 2012). Briefly, the directed migration of the deep layer cells (deep cells) toward the midline of the non-neural epithelium which underlies the superficial cells assists in achieving complete closure of the tube. The movement of these highly motile deep cells is assumed to pull the overlying superficial cells through cell-to-cell interactions, eventually bringing the two layers of cells to the midline (Morita et al. 2012). To simulate deep cell migration toward the midline, the five cells at the edge of the deep layer (light brown-colored cells in Fig. 1a) underwent cell migration toward the midline in response to migration energy Eq. (8). Our model expresses the medial movement of the deep cells pulling the superficial layer, instead of the molecular basis for medial migration of the cell. Because medial displacement of the superficial layer begins at mid-neurulation (Wallingford and Harland 2002), the cell migration energy was imposed beginning at time $t = 0.49$.

### Characteristic geometrical parameters

To quantify the tissue deformations, we define geometrical parameters characterizing both the tissue and cell shapes. Figure 1b shows definitions of the parameters on the tissue scale. A gap distance, $D_\mathrm{gap}$, quantifies the mean end-to-end distance between the two centroids of the apical surfaces of the lateral boundary cells of the neural plate, indicating the degree of tube closure completion. The tissue height and width of the neural plate are defined by the difference between the largest and smallest *z*- and *x*-components of the position vector of the neural cell centroid. The tissue height/width ratio, $\Lambda _\mathrm{{hw}}$, is then defined as the tissue height divided by the width.

The cell shape is quantified by two parameters: the apical/basal width ratio, $\lambda ^\mathrm{{ab}}$, and the cell height/width ratio, $\lambda ^\mathrm{{hw}}$, of the cell. $\lambda ^\mathrm{{ab}}$ is defined as the mean value of the apical width divided by the basal width of the superficial neural cell and thus indicates the degree of asymmetry in the cell shape along the AB axis. $\lambda ^\mathrm{{hw}}$ is defined as the mean value of the height divided by the larger of the apical or basal side widths of the superficial neural cell and thus indicates whether the cell adopts a flat or columnar shape. To quantify the cell width of an arbitrary shape, we calculate an equivalent apical (basal) width from a regular hexagon with an area equal to the apical (basal) area of the cell.

### Materials and methods in experiments

Experiments with *X. laevis* embryos were performed essentially as previously described (Suzuki et al. 2010). To inhibit the functions of MID1/2 and Shroom3 proteins, morpholino antisense oligonucleotides (Mo; Gene Tools) against MID1/2 (Suzuki et al. 2010) and Shroom3 (Haigo et al. 2003) were injected into the dorsal side of four-cell embryos at 4–6 pmol per embryo with 100 pg EGFP mRNA as a tracer. The embryos were fixed at stage 16 for the double inhibition experiment. For the MID1/2 inhibition experiment, the control embryos were fixed at stage 19 and the MID-Mo-injected embryos at stage 21, because the completion of neural tube closure was delayed by MID1/2 inhibition. Quantitative analyses of the cellular morphology were performed as previously described (Suzuki et al. 2010). The tissue morphologies were manually determined using ImageJ software (NIH) (Schneider et al. 2012).

## Results

### Apical constriction, a tissue autonomous process

**Fig. 2:**
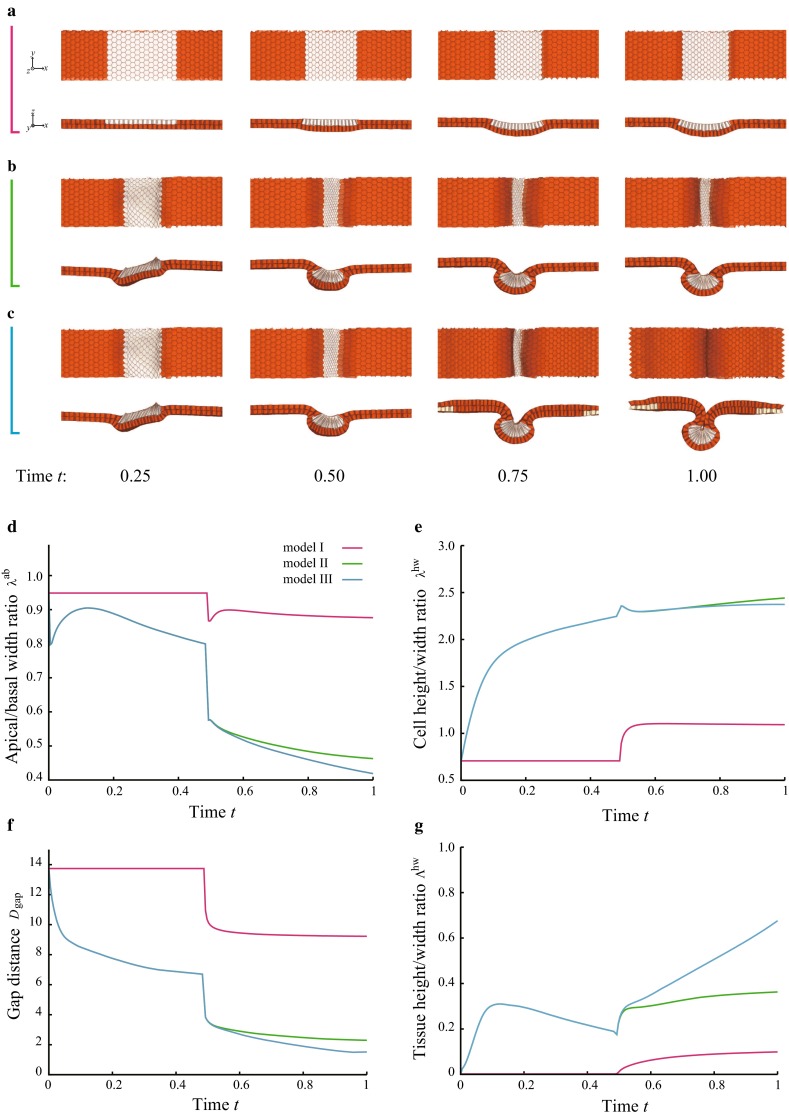
Different combinations of the physical events during neural tube closure examined using **a** model I, **b** model II, and **c** model III are shown as snapshots over time. The migrating cells are outside of the visualized area except $t=0.75$ and 1.0 in the panel (**c**). **d** The apical/basal width ratio, $\lambda ^\mathrm{{ab}}$, of the superficial neural cells as a function of time *t*. **e** The cell height/width ratio, $\lambda ^\mathrm{{hw}}$, of the superficial neural cells as a function of time *t*. **f** The gap distance, $D_\mathrm{gap}$, as a function of time *t*. **g** The tissue height/width ratio, $\Lambda ^\mathrm{{hw}}$, as a function of time *t*

First, we determined the importance of apical constriction using model I, which excludes the effects of cell elongation and migration. Figure 2a shows snapshots of the model I simulation (see also Supplementary Movie 1). Beginning at time $t=0.49$, apical constriction acted on all of the superficial neural cells, which are displayed in white in the figure. These apically constricted cells adopted a slightly wedge-like shape with an apical/basal width ratio, $\lambda ^\mathrm{{ab}}$, of approximately 0.9 after the application of apical constriction (Fig. 2d). Figure 2e shows the height/width ratio, $\lambda ^\mathrm{{hw}}$, of these constricted cells. Because the cell volume was constrained by the volume elastic energy, apical constriction resulted in cell elongation that compensated for the volume reduction caused by shrinkage of the apical surface area.

Consequently, the neural plate was slightly deformed to a basally convex shape, indicating an invagination. Figure 2f shows the gap distance, $D_\mathrm{gap}$, over time, which decreased slightly after the application of apical constriction. Figure 2g shows the tissue height/width ratio $\Lambda _\mathrm{{hw}}$ of the neural plate over time, which also shows that the depth of invagination was very small. Thus, apical constriction actually induced the invagination, but that induction was insufficient to achieve complete neural tube closure.

### Cell elongation associated with apical constriction

We next incorporated cell elongation in addition to apical constriction (model II) and examined how cell elongation and thickening of the plate contribute to neural tube closure. Figure 2b shows snapshots of the model II simulation (see also Supplementary Movie 2). Because the cell volume was constrained by the volume elastic energy, cell elongation resulted in a reduction in the lateral width of the cell. This reduction in cell width is theoretically independent of AB polarity; however, because the basal surfaces of the superficial cells are in contact with deep cells, they are more difficult to deform than the apical surfaces, resulting in a $\lambda ^\mathrm{{ab}} < 1$ (Fig. 2d). These neural cells approximately doubled in height prior to the application of apical constriction at time $t=0.49$ (Fig. 2e), reproducing the trend of cell height change observed in vivo (Lee et al. 2007).

Apical constriction, applied to the superficial neural cells at time $t=0.49$, further decreased the apical surface specifically, and the basal surface subsequently expanded to maintain a constant cell volume (Fig. 2d). This also slightly increased the cell height (Fig. 2e). Thus, these elongated cells became apically narrow wedge-like shapes, leading to geometric rounding of the neural plate.

As shown in Fig. 2f, the gap distance, $D_\mathrm{gap}$, was significantly decreased compared with the result from model I. Even before the application of apical constriction, $D_\mathrm{gap}$ was decreased in model II because the lateral width of the cell was decreased by the cell elongation. Just after the onset of apical constriction ($t = 0.49$), $D_\mathrm{gap}$ was sharply decreased. Apical constriction rapidly changed the cell shape to a wedge in our simulation. Therefore, the folding of the tissue into a round shape also progressed rapidly, leading to the sharp decrease in $D_\mathrm{gap}$. This discontinuity in the time variation may have resulted from the 3D vertex expressions of the multiple cells, but does not affect the causative relationship between the sharp decrease in $D_\mathrm{gap}$ and the combination of apical constriction and cell elongation.

After time $t = 0.8$, $D_\mathrm{gap}$ remained nearly constant. Thus, cell elongation drove neural plate folding and tube closure by increasing the magnitude of tissue deformation. However, neural tube closure was not completed, as a gap of approximately two cells remained, even when the simulation was run for twice the closure duration obtained using model III (control).

Previously, Morita *et al.* reported that the inhibition of deep cell migration caused incomplete neural tube closure, leaving a slit along the dorsal midline (Morita et al. 2012). The shape of the neural plate observed in the simulation by model II qualitatively coincided with the defect observed in the experiment.

### Migration of deep cells, a non-tissue autonomous process

We incorporated the medial movement of the cells, along with apical constriction and cell elongation, in model III. Figure 2c shows snapshots from the simulation using model III that resulted in complete neural tube closure (see also Supplementary Movie 3). Because the behavior of the ratios $\lambda ^\mathrm{{ab}}$ and $\lambda ^\mathrm{{hw}}$ over time in model III was nearly the same as those in model II (Fig. 2d,e), the two models produced almost the same cell shape change. This result indicates that cell migration has a negligible contribution to cell deformation. However, the gap distance, $D_\mathrm{gap}$, in model III continued to decrease with time, whereas it stopped decreasing in model II (Fig. 2f). The tissue height/width ratio, $\Lambda ^\mathrm{{hw}}$, also increased markedly with time compared with that predicted in model II (Fig. 2g). Thus, cell migration promoted tissue displacement rather than cell deformation, suggesting that cell migration provides the necessary final push on the tissue scale to accomplish neural tube closure.

### Perturbation simulations and experiments

To further address how cell elongation, cell migration, and their cooperation with apical constriction contribute to neural tube closure, we performed perturbation simulations by (i) inhibition of both apical constriction and cell elongation (AC/EL inhibition) and (ii) inhibition of cell elongation alone (EL inhibition). In addition to determining whether the simulation models presented in the preceding sections recapitulated the neural tube closure observed in vivo, the predicted effects after in silico perturbations were confirmed experimentally.

#### Inhibition of both apical constriction and cell elongation

**Fig. 3:**
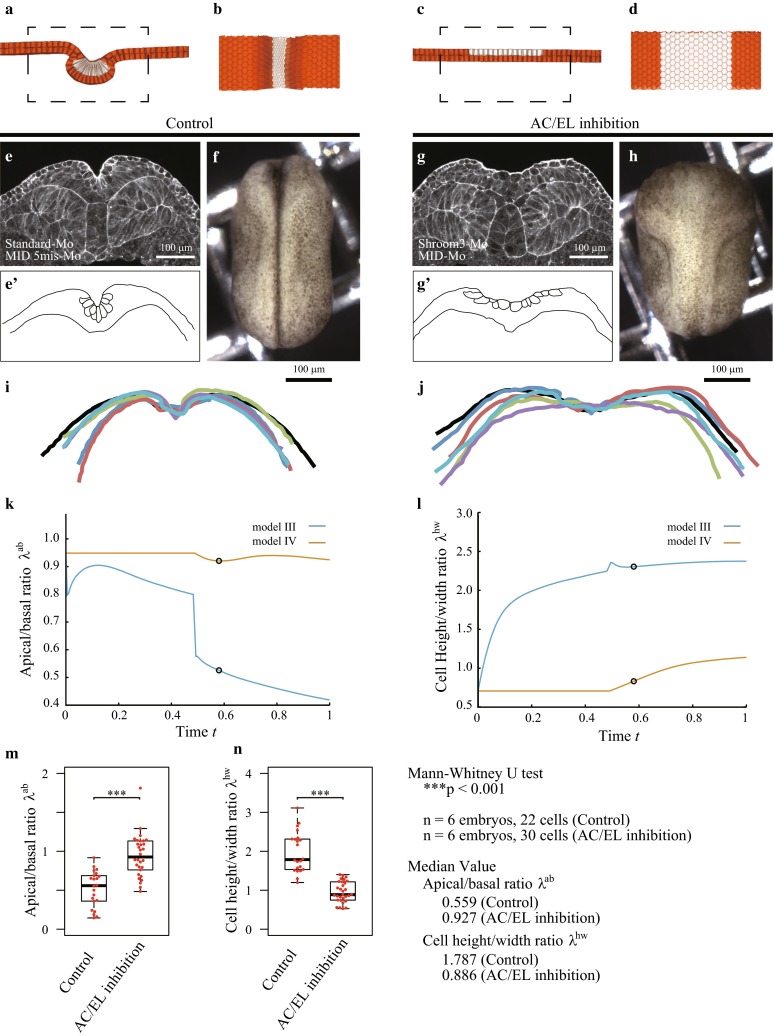
Shapes of control, and MID1/2 and Shroom3 inhibited (AC/EL inhibition) embryos observed in silico and *in vivo*. Mediolateral cross-sectional views of the neural tissue shape at time $t=0.58$ in the simulations using **a** model III (control) and **c** model IV (AC/EL inhibition). **b**, **d** Dorsal side views at the dashed rectangular regions of (**a**, **c**). **e**, **g** The neural tissue and cells in vivo were observed by phalloidin staining (F-actin) at stage 16. **f**, **h** Bright field image of overall shape of the entire embryo at stage 16. The outlines **e**’, **g**’ show the neural tissue and cells, while *i*, *j* show the apical surface of the neural epithelial tissues, where six lines are obtained from six embryos. Comparisons between models III and IV show **k** the apical/basal width ratio, $\lambda ^\mathrm{{ab}}$, and **l** the cell height/width ratio, $\lambda ^\mathrm{{hw}}$, as functions of time. The *circles mark* the values at $t=0.58$, which are compared with the experimental values. **m**, **n** The experimental values of those ratios at stage 16

We inhibited apical constriction and cell elongation in model IV, where we exclude the apical circumferential elastic energy and the cell height elastic energy, and assessed the contribution of deep cell movement. The neural plate retained a nearly flat shape in model IV (Fig. 3a–d and Supplementary Movie 4). Because of the lack of apical constriction in model IV, the cell shape retained an almost cuboidal shape ($\lambda ^\mathrm{{ab}}\sim 1$) throughout the simulation (Fig. 3k). The deep cell migration pulled the overlying superficial cells toward the midline, causing them to press against the superficial neural cells, which resulted in a reduction in the width of the neural cells. Then, because of the cell volume constraint, the height of the neural cells was increased (an increase in $\lambda ^\mathrm{{hw}}$ in Fig. 3l).

This condition in model IV was reproduced experimentally by inhibiting the functions of MID1 and MID2, both of which are proteins required for cell elongation (Suzuki et al. 2010), and Shroom3, a protein required for both cell elongation and apical constriction (Haigo et al. 2003; Lee et al. 2007) (AC/EL inhibition). Figure 3e–j demonstrates that the neural plate in the embryos with AC/EL inhibition was flat, which resembled the tissue morphology predicted by the simulation using model IV. In addition, the cell morphological changes measured experimentally coincided with those observed in the simulation. The changes in the apical/basal ratio $\lambda ^\mathrm{{ab}}$ ranged from 0.53 (model III) to 0.92 (model IV) in the simulations (at time $t=0.58$ in Fig. 3k) and from 0.56 (control) to 0.93 (AC/EL inhibition) experimentally (Fig. 3m). The change in $\lambda ^\mathrm{{hw}}$ ranged from 2.3 (model III) to 0.83 (model IV) in the simulations (at time $t=0.58$ in Fig. 3l) and from 1.8 (control) to 0.89 (AC/EL inhibition) experimentally (Fig. 3n). These results suggest that the model fairly represented the cell deformations of real embryos.

#### Inhibition of cell elongation

**Fig. 4:**
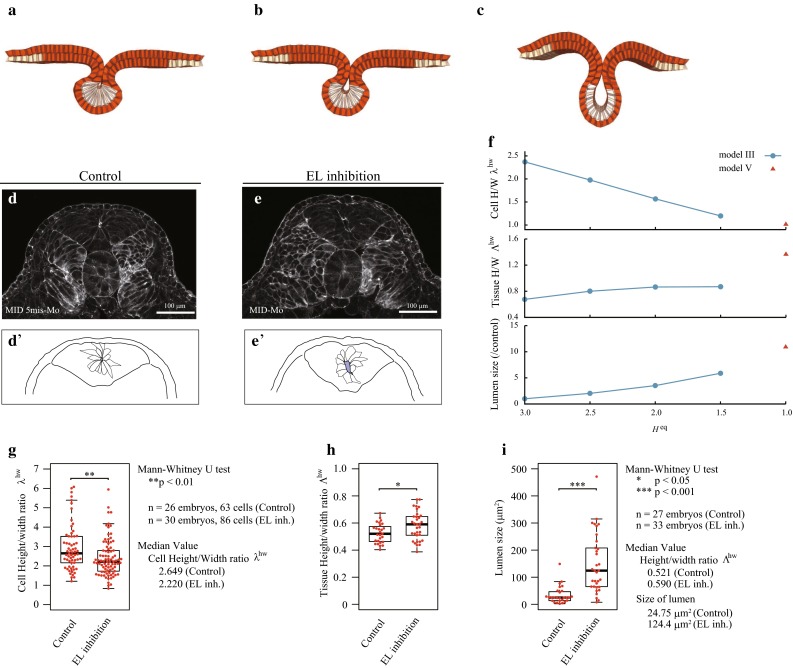
Shapes of control, and MID1/2 inhibited (EL inhibition) embryos observed in silico and in vivo. The neural tube closure in the simulations using **a** model III with $h^\mathrm{{eq}}=3.0$ (control), **b** model III with $h^\mathrm{{eq}}=2.5$ (weak EL inhibition) and **c** model V with $h^\mathrm{{eq}}=1.0$ (complete EL inhibition). **d**, **e** Phalloidin staining (F-actin) at stages 19 for control and 21 for EL inhibition experiments. **d**’, **e**’ Outlines of neural tissues and cells. The solid-filled area indicates the lumen. **f** (from *top* to *bottom*) the cell height/width ratio $\lambda ^\mathrm{{hw}}$, tissue height/width ratio $\Lambda ^\mathrm{{hw}}$, and lumen size (normalized by that of the control) in simulations. Comparisons between the control and EL inhibition embryos for **g** the cell height/width ratio, $\lambda ^\mathrm{{eq}}$, **h** the tissue height/width ratio, $\Lambda ^\mathrm{{hw}}$, and **i** the lumen size in experiments

To investigate the effects of cell elongation, we examined simulations in which cell elongation is gradually inhibited, but apical constriction and cell migration both remain present. We assigned $h^\mathrm{{eq}}$ to be each of {1.5, 2.0, 2.5} in model III for expressing weak inhibition of cell elongation and excluded the height elastic energy to express complete inhibition of cell elongation (model V). Figure 4a–c shows snapshots of the control, weak EL inhibition, and complete EL inhibition at neural tube closure (see also Supplementary Movies 5 and 6). Comparing the results obtained by the control and EL inhibition models, apical constriction and cell migration were sufficient to create a tube-like structure with closure, suggesting that cell elongation was not necessary. However, the geometry of the tube structure in the complete EL inhibition model was much different from that observed in the control.

The tissue height/width ratio $\Lambda ^\mathrm{{hw}}$ and lumen size, which is normalized to that at the control, both increase as the cell height/width ratio $\lambda ^\mathrm{{hw}}$ decreases (Fig. 4f). Cell elongation actually reduces the lumen size because the width of the tissue at the apical end is a geometrical constraint on the lumen size, with smaller widths constricting it. Thus, the decrease in $\lambda ^\mathrm{{hw}}$ caused lumen opening.

This EL inhibition was reproduced experimentally by inhibiting the functions of the MID proteins. Figure 4d–e’ shows neural tube closure with a large-sized lumen in EL inhibition embryos as predicted by the simulations. The cell morphological change coincided with those obtained in the weak EL inhibition at $h^\mathrm{{eq}}$ = 2.5, rather than those obtained by model V. The change in the cell height/width ratio $\lambda ^\mathrm{{hw}}$ ranged from 2.4 (control) to 2.0 (EL inhibition at $h^{\mathrm{eq}}$ = 2.5) in the simulations (Fig. 4f), and from 2.6 (control) to 2.2 (EL inhibition) experimentally (Fig. 4g). The inhibition of the functions of the MID proteins actually suppressed cell elongation, but the magnitude of the suppression was not strong as those observed by inhibition of the functions of both MID proteins and Shroom3. The tissue morphological changes also show the same trend as those in the simulations (at $h^\mathrm{{eq}}=$ 2.5 relative to the control in Fig. 4f). In the experiments, $\Lambda ^\mathrm{{hw}}$ was approximately 1.1-fold higher than that in the control embryos (Fig. 4h), and 1.2-fold higher than in the simulations (Fig. 4f). The lumen size was approximately fivefold larger than in the experimental control (Fig. 4i) and twice the size of that in the simulations (Fig. 4f). These slight quantitative differences may arise from the simplification of the underlying layer and from neglecting the viscoelasticity of the surrounding matrix and hydrostatic pressure in the enclosed embryos.

**Fig. 5:**
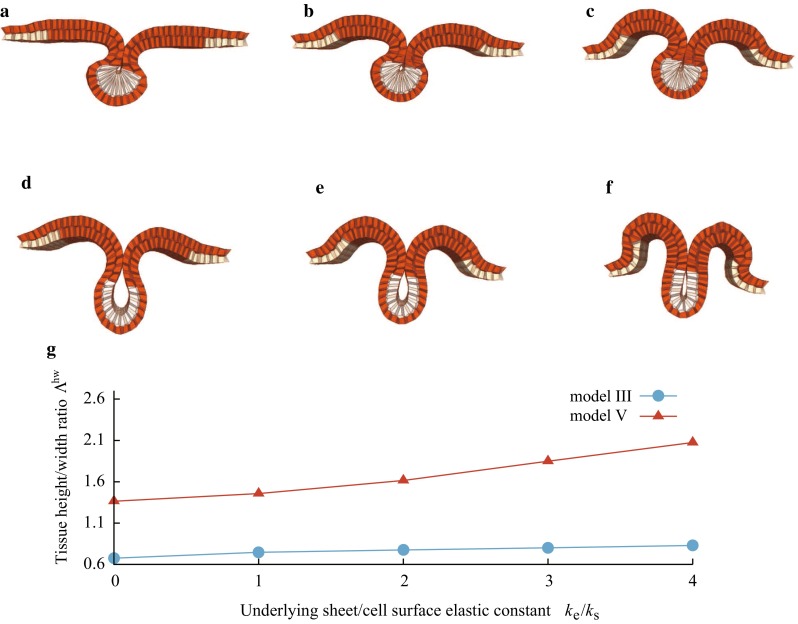
Effect of the elastic sheet underneath the deep layer on neural tube shapes were examined with and without cell elongation. Simulation results using model III (control) for **a** $k_e/k_s$ = 0, **b** $k_e/k_s$=2, **c** $k_e/k_s$=4, and using model V (EL inhibition) for **d** $k_e/k_s$=0, **e** $k_e/k_s$=2, **f** $k_e/k_s$=4, in which $k_e/k_s$ is the sheet elastic constant $k_e$ normalized by the cell surface elastic constant $k_s$. **g** Tissue height/width ratio, $\Lambda ^\mathrm{{hw}}$, as a function of $k_e/k_s$

**Fig. 6:**
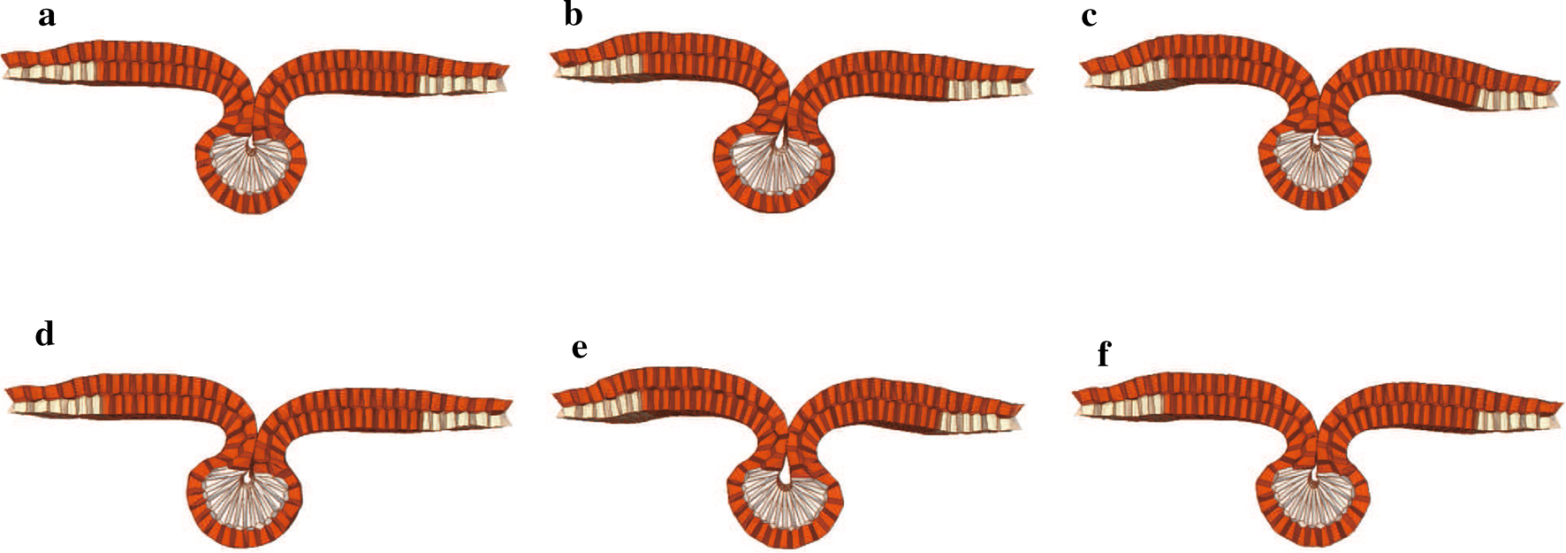
Effect of permutation of onset time of the apical constriction (AC), cell elongation (EL), and cell migration (CM) on the neural tube shape. The order of three events is **a** AC $\rightarrow$ EL $\rightarrow$ CM, **b** EL $\rightarrow$ CM $\rightarrow$ AC, **c** CM $\rightarrow$ AC $\rightarrow$ EL, **d** EL $\rightarrow$ AC $\rightarrow$ CM, **e** CM $\rightarrow$ EL $\rightarrow$ AC, **f** AC $\rightarrow$ CM $\rightarrow$ EL. The onset time of the first, second, and third events is $t=$ 0.00, 0.25, and 0.49, respectively

Interestingly, despite these simplifications, the control model showed good agreement in geometrical parameters ($\Lambda ^\mathrm{{hw}}$, $\lambda ^\mathrm{{ab}}$, $\lambda ^\mathrm{{hw}}$) with the experimental measurements of control embryos, bringing a hypothesis that cell elongation might play a role in protecting tube closure from potential disturbances from the surrounding mechanical environment.

To test this hypothesis, we simulated neural tube closure using model III (control) and V (complete EL inhibition) with an additional elastic sheet underneath the deep layer. We supposed the lateral edges of the elastic sheet were fixed at their initial positions to perturb invagination of the neural plate. Then, the energy function, $u^\mathrm{{e}}$, of the underlying sheet with the elastic constant $k_e$ was introduced by $u^\mathrm{{e}}=k_e(A/A_0-1)^2/2$, where the sheet area *A* was defined by the sum of the base area of the deep layer and the effective area that was passed over by medial movements of the deep cell at most ends, which at time $t=0$ was assigned as the reference area, $A_0$.

Figure 5 shows snapshots of neural tube closure obtained by models III and V (see also Supplementary Movies 7 and 8). The neural tube in simulations using model III were almost the same shape as each other, regardless of the sheet elasticity. In contrast, in simulations using model V, increases in sheet elastic constant resulted in an increase in the tissue height/width ratio and closure of the lumen.

Although the current simulations represent only a part of the mechanical effects arising from the surrounding environment, these results supported the hypothesis. Thus, we anticipate that cell elongation-mediated amplification of tissue deformation may assist in protecting tube closure from the mechanical effects of the surrounding environment, thus ensuring the robustness of the final shape of the neural tube in *Xenopus*.

### Permutation of three events

Because our study relies on computer simulations, we can assign an arbitrary onset time to each cellular event. Therefore, in addition to inhibition of cell morphogenetic events by previous sections, we examined whether neural tube closure would be affected by the order of the three events of apical constriction, cell elongation, and cell movement.

Figure 6 shows simulation results of neural tube shapes at closure. From the results, the completion of neural tube closure could be observed in all possible permutations of the three events. Slightly large lumen sizes, although still smaller than that in EL inhibition, were obtained if the onset time of apical constriction was last to occur (Fig. 6b, e); however, the shapes of the neural tube were completely insensitive to the order of the events compared with those obtained by inhibition models. Thus, during neural tube closure, it might simply be important that all three events are ultimately activated regardless of the order.

## Discussion

We investigated the physical roles that apical constriction, cell elongation, and cell migration play during neural tube closure using 3D vertex simulations. We confirmed that apical constriction cued the bending of the neural plate by pursing the apical surface of the neural cells. Neural cell elongation in concert with apical constriction further narrowed the apical surface of the cells because of the cell volume constraint, enabling the rapid folding of the neural plate. Migration of deep cells provides the additional tissue deformation necessary to achieve complete neural tube closure.

One unique developmental feature of amniotes is the characteristic morphology of their early neural tube: The lumen opening is more evident and is formed deeper from the dorsal side (Harrington et al. 2009). It is intriguing to note that the morphology obtained in our simulation without cell elongation (model V) resembled that of the amniote neural tube in its roomy opening and larger tube length along the DV axis in the cross-sectional view (Fig. 4c). This resemblance also demonstrates the power of mathematical modeling to provide physical insights into relationships between variations in organ- and cell-level morphologies.

In this series of models, we did not consider hinge points, the small areas in which apical constriction initially and preferentially occurs. In the anterior region of the *Xenopus* neural tube, there are three hinge points in a single cross section of the embryo, and the hinge points are continuous along the AP axis to allow the zipping up of the neural tube. Thus, our models could be improved by defining specific cells in the area corresponding to the hinge points that cause apical constriction. We speculate that these hinge points may be required to fold a wide neural plate as described below.

Previous quasi-3D simulations (Clausi and Brodland 1993) showed that a neural plate undergoing cell elongation in concert with apical constriction did not fold as much as was observed in our results. This difference is likely caused by the difference in the number of cells composing the neural plate used in each model, with the number of cells in our model being almost half of that used by Clausi and Brodland (1993). The difference in the number of cells in the neural plate depends on the difference in the ML size of the neural plate in animal models and also on the region of interest selected from the neural plate along the AP axis. In a wide neural plate along the length of the ML axis, the neural plate was relatively flat because a balance between the apical forces except at the boundary between the neural and non-neural cells, where the neural plate was rounded (Clausi and Brodland 1993). As a result, strong hinge points at the midline and boundary (dorsolateral positions) were required for neural plate folding (Clausi and Brodland 1993). In the present study, the size of the neural plate appeared to be small enough for the plate to round from the boundary to the midline.

Finally, there are several potential limitations to our models and simulations. As shown in Fig. 2b and c, cell elongation acting alone resulted in tissue bending sharply into three apparent regions (snapshots at time $t= 0.25$ in models II and III), while this sharp bending transiently appeared and almost flattened over the apical surface before the onset of apical constriction and cell migration (see also Supplemental Movies 2 and 3). Because of the deep layer on the basal side of the superficial cells, the effective stiffness of the apical surface of the superficial neural cells is different to that of the basal surface. This mechanical anisotropy caused bending of the tissue when cell elongation acted alone. In fact, excluding the deep layer in the simulation, cell elongation alone did not induce bending of the tissue, but only induced thickening of the neural plate (Supplementary Movie 9). Thus, the boundary condition on the apical side may be important for investigating the tissue deformation induced by cell elongation prior to apical constriction.

**Fig. 7:**
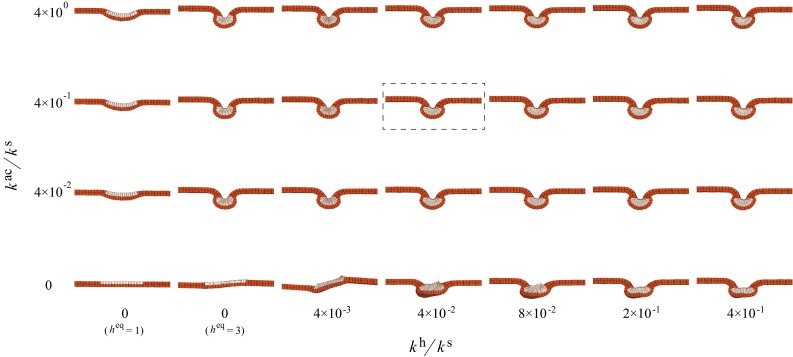
Cell and tissue shapes at time $t=1$ for the two ratios, $k^\mathrm{{h}}/k^\mathrm{{s}}$ and $k^\mathrm{{ac}}/k^\mathrm{{s}}$. The parameters in the control model are given by the dashed box. $h^\mathrm{{eq}}=1$ and 3 are tested at $k^\mathrm{{h}}/k^\mathrm{{s}} = 0$, while $h^\mathrm{{eq}}=3$ for $k^\mathrm{{h}}/k^\mathrm{{s}} > 0$

Because we adopted a periodic boundary condition on the AP axis, the neural plate was restrained from changing its length along that axis in the simulations. Accordingly, the neural cells were deformed mainly along the ML axis, even though the isotropic apical constriction acted on the apical surface, as defined in Eq. (7). Anisotropic constriction caused an anisotropic deformation of the apical surface of the cell, resulting in bending of the neural plate to induce a furrow, not a round pit, whereas anisotropic apical constriction may be mediated by planar cell polarity (PCP) (Nishimura et al. 2012; Ossipova et al. 2015). By virtue of this periodic boundary condition, our simulations resembled the anisotropic deformation of neural cells. However, the convergent extension of the neural plate (Davidson and Keller 1999), which was also related to PCP (Wallingford and Harland 2002; Ueno and Greene 2003), was not recapitulated in the present study because dynamic cell intercalation along the AP axis was suppressed by the periodic boundary condition. Also, because we did not model any molecular basis for the deep cell migration, we could not examine the mechanisms by which deep cells migrate toward the midline in the 3D embryo. Real embryos are closed systems, and the fields of cells that make up tissues and undergo remodeling are always limited. Thus, to refine our models to recapitulate the tissue dynamics of embryos more accurately, we could use boundary conditions similar to those found in embryos by accounting, for example, for their shapes and force balance.

### Electronic supplementary material

Below is the link to the electronic supplementary material.

Supplementary material 1 (mp4 418 KB)

Supplementary material 2 (mp4 809 KB)

Supplementary material 3 (mp4 903 KB)

Supplementary material 4 (mp4 567 KB)

Supplementary material 5 (mp4 1931 KB)

Supplementary material 6 (mp4 904 KB)

Supplementary material 7 (mp4 1202 KB)

Supplementary material 8 (mp4 2475 KB)

Supplementary material 9 (mp4 220 KB)

Supplementary material 10 (docx 12 KB)

## Appendix: Parameter survey

To investigate the effect of altering each constant on cell and tissue shapes, we obtained final shapes without deep cell migration (external forces) for a range of cell height and apical circumferential ring elastic constants against the cell surface elastic constant, $k^\mathrm{{h}}/k^\mathrm{{s}}$ and $k^\mathrm{{ac}}/k^\mathrm{{s}}$, respectively. Here, we explain the reason why we have chosen these two ratios as follows. The energy function *U* of the cell shape is rewritten as

9 $$\begin{aligned} U= & {} \sum _\mathrm{{cell}} \frac{1}{2}k^\mathrm{{v}}\left[ \left( \frac{v}{v^\mathrm{{eq}}} - 1 \right) ^2 + \frac{k^\mathrm{{s}}}{k^\mathrm{{v}}}\left\{ \left( \frac{s}{s^\mathrm{{eq}}} - 1 \right) ^2 \right. \right. \nonumber \\&+ \left. \left. \frac{k^\mathrm{{h}}}{k^\mathrm{{s}}}\left( \frac{h}{h^\mathrm{{eq}}} - 1\right) ^2 + \frac{k^\mathrm{{ac}}}{k^\mathrm{{s}}}\left( \frac{l}{l^\mathrm{{eq}}} - 1\right) ^2 \right\} \right] . \end{aligned}$$

Because cells maintain a constant volume, the volume elastic constant $k^\mathrm{{v}}$ should be assigned as the highest value in these constants. To consider the cell shape change simply, the cell volume is assumed to be always kept constant. Thus, the first term on the right-hand side in Eq. (9) can be omitted from the consideration, and the balance among the last three terms in the bracket $\{ \cdot \}$ becomes the most important in cell shaping, where the ratios of $k^\mathrm{{h}}/k^\mathrm{{s}}$ and $k^\mathrm{{ac}}/k^\mathrm{{s}}$ are the only elastic constants in the bracket.

The simulation results in Fig. 7 show cell and tissue shapes at time $t=1$, where the result enclosed by the dashed rectangle indicates the control value. These shapes are not sensitive to the values of these ratios unless either of the ratios is zero. Because the deep layer cells are almost incompressible, cell elongation causes preferential reduction in the apical area of the superficial neural cell to maintain the cell volume. Therefore, as $k^\mathrm{{h}}/k^\mathrm{{s}}$ increases, the neural plate shows an invagination, even without the apical constriction. A similar numerical result has been also reported by quasi-3D simulations (Clausi and Brodland 1993), where the transverse forces along the AB axis caused invagination if the incompressive base was applied at the basal side. However, when we further imposed the cell migration energy, the lumen opening was realized with a higher value of $k^\mathrm{{h}}/k^\mathrm{{s}}$, which was not the case of the experiment.

Based on the assumption that the cell was a columnar shape with a volume $v^\mathrm{{eq}}$ and a height $h^\mathrm{{eq}}$ at equilibrium, the reference surface area was expressed by the reference cell volume and surface (Table 1). By assigning $h^\mathrm{{eq}}=3$ under apical constriction ($k^\mathrm{{ac}}/k^\mathrm{{s}}\ne 0$), cells were elongated without the cell height elastic energy ($k^\mathrm{{h}}/k^\mathrm{{s}}=0$). Thus, the cell height elastic energy is redundant to the cell surface elastic energy under apical constriction; however, cell elongation was not observed without apical constriction ($k^\mathrm{{ac}}/k^\mathrm{{s}} = 0$). In the experiment (Lee et al. 2007), cell elongation occurs prior to apical constriction, indicating the requirement of the cell height elastic energy independently.
