# Supplementary material for: Mechanical roles of apical constriction, cell elongation, and cell migration during neural tube formation in Xenopus
Source: Biomech Model Mechanobiol. 2016 May 18;15(6):1733–46. doi: 10.1007/s10237-016-0794-1 (PMC5106510; doi:10.1007/s10237-016-0794-1)
Supplement: Supplementary file 10 — Supplementary material 10 (docx 12 KB) [file 10237_2016_794_MOESM10_ESM.docx]

Movie1: Inhibition of cell elongation and cell migration (model I)

Movie2: Inhibition of cell migration (model II)

Movie3: Control model (model III)

Movie4: Inhibition of apical constriction and cell migration (model IV)

Movie5: Inhibition of cell elongation (model V)

Movie6: Weak inhibition of cell elongation (model III with *h*^eq^=2.5)

Movie7: Examination of the basement elasticity on neural tube formation using the control model (model III: *k*_e_/*k*_s_ = 4)

Movie8: Examination of the basement elasticity on neural tube formation using the cell elongation inhibition model (model V: *k*_e_/*k*_s_ = 4)

Movie9: Examination of monolayer neural plate with cell elongation
